# Nevus Comedonicus with Epidermolytic Hyperkeratosis: A Case Report

**DOI:** 10.30699/IJP.2022.542761.2767

**Published:** 2022-03-08

**Authors:** Faezeh Taghavi, Sima Davoodi, Naser Tayyebi Meibodi, Yalda Nahidi, Mostafa Izanlu

**Affiliations:** 1 *Department of Dermatology, Imam Reza Hospital, Mashhad University of Medical Sciences, Mashhad, Iran*; 2 *Department of Pathology, Imam Reza Hospital, Mashhad University of Medical Sciences, Mashhad, Iran*

**Keywords:** Epidermolytic hyperkeratosis, Nevus comedonicus, Rare skin disorders

## Abstract

Nevus comedonicus (NC) is a rare developmental anomaly of the folliculosebaceous apparatus, which appears as numerous dilated papules containing firm, darkly pigmented, horny plugs. It appears shortly after birth and mostly before the age of 10; however, late-onset cases have been reported. There is no gender or racial predilection. Moreover, NC can be a component of nevus comedonicus syndrome, a neurocutaneous disorder with skeletal, ocular, and central nervous system abnormalities. EHK properties in NC are not a common finding and are rarely seen in association with each other. This paper reports a healthy, 27-year-old young woman who has been developing numbers of asymptomatic unilateral linear skin lesions on her chest, waist, right thigh, and popliteal fossa in a unilateral linear pattern over ten years. Skin biopsy revealed dilated follicular ostia with orthokeratotic hyperkeratosis, columns of parakeratosis, cornoid flagellation, epidermolytic hyperkeratosis, and mild acanthosis on its wall.

## Introduction

Nevus comedonicus (NC) is an uncommon cutan-eous disease characterized by dilated follicular openings with dark cornified material like classical comedones (1). Epidermolytic hyperkeratosis (EHK) is an incidental and rare finding in NC (2).

Here authors report a case with these properties in histopathology.

## Case Report

**Fig. 1:**
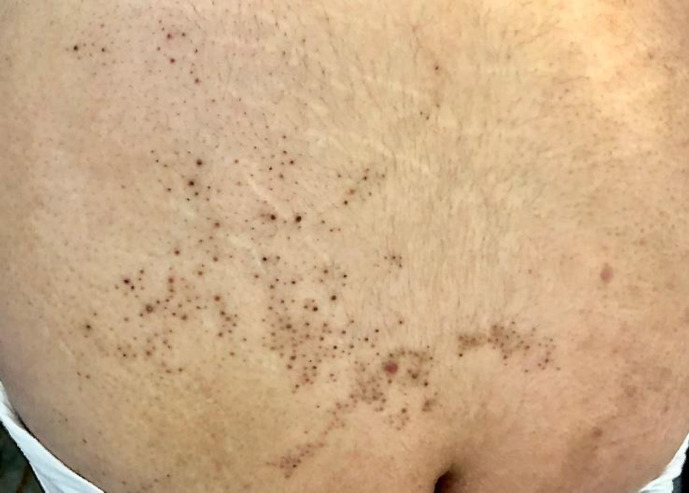
Black-brown horny follicular papules on the waist

**Fig. 2:**
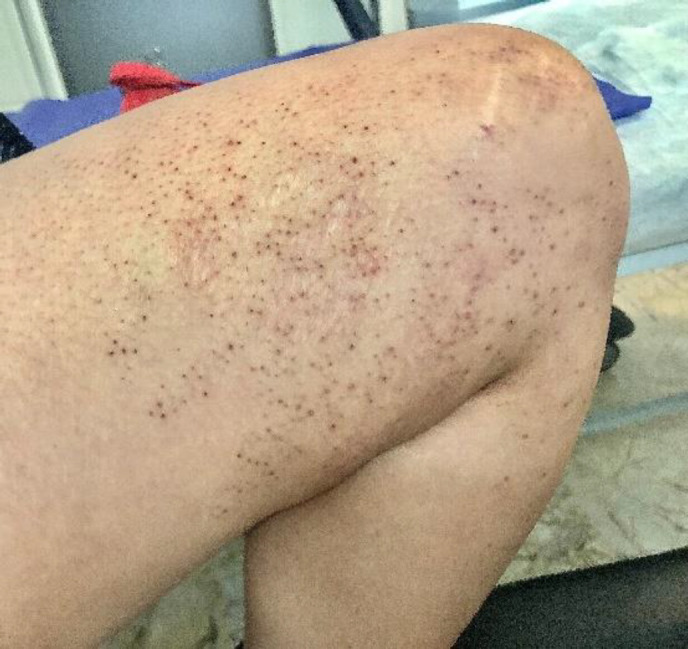
Keratotic follicular comedo-like papules on the right thigh

A 27-year-old young woman was referred to our clinic with plenty of asymptomatic skin lesions deve-loped over ten years. Although her family history was negative, she had clusters of black-brown horny folli-cular papules on her chest, waist, right thigh, and popliteal fossa in a unilateral linear pattern (Figures 1 and 2). Additionally, the lesions were steady in size and number over time. The patient was otherwise healthy with no systemic complaints. 

Based on the clinical examination and the patient's history, several differential diagnoses were made, incl-uding nevus comedonicus, lichen planopilaris, pityri-asis rubra pilaris (PRP) as well as lichen spinulosus, porokeratotic eccrine ostial, and dermal duct nevus (PEODDN).

Therefore, a biopsy was performed to definitively diagnose the lesion.

**Fig. 3:**
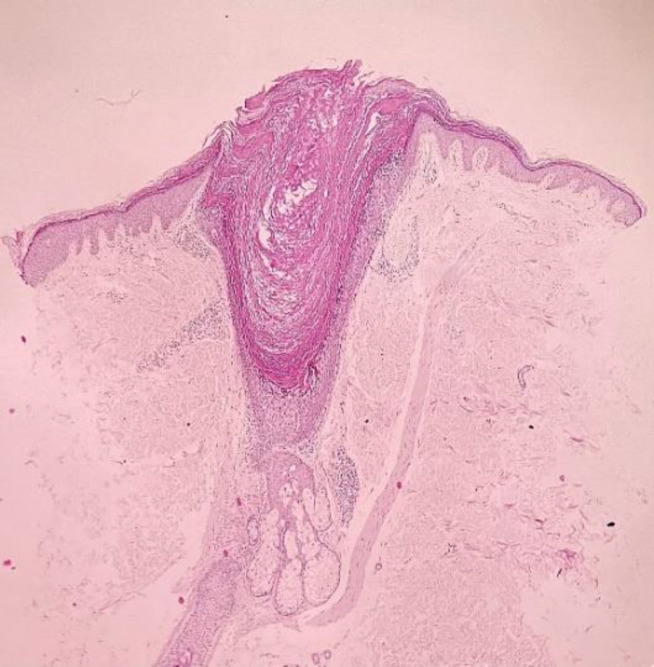
Dilated follicular ostia with orthokeratotic hyperkeratosis

**Fig. 4:**
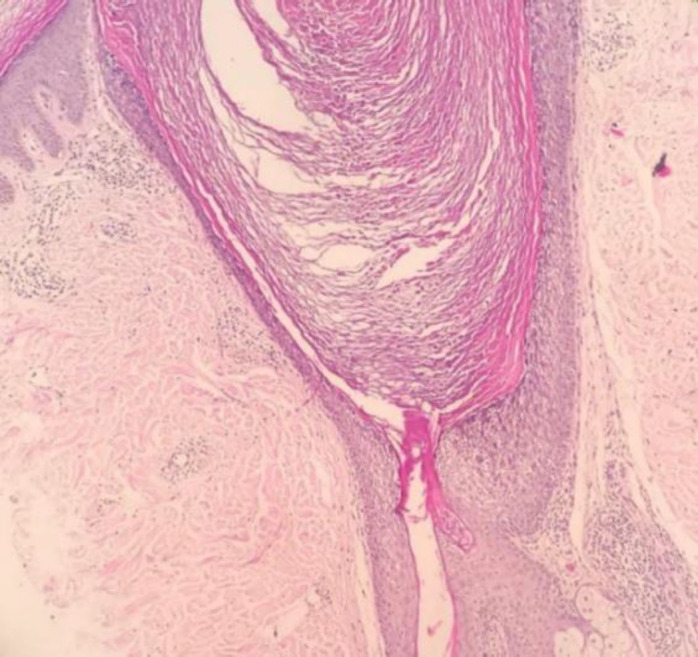
Comedo-like openings in follicular ostia

**Fig. 5:**
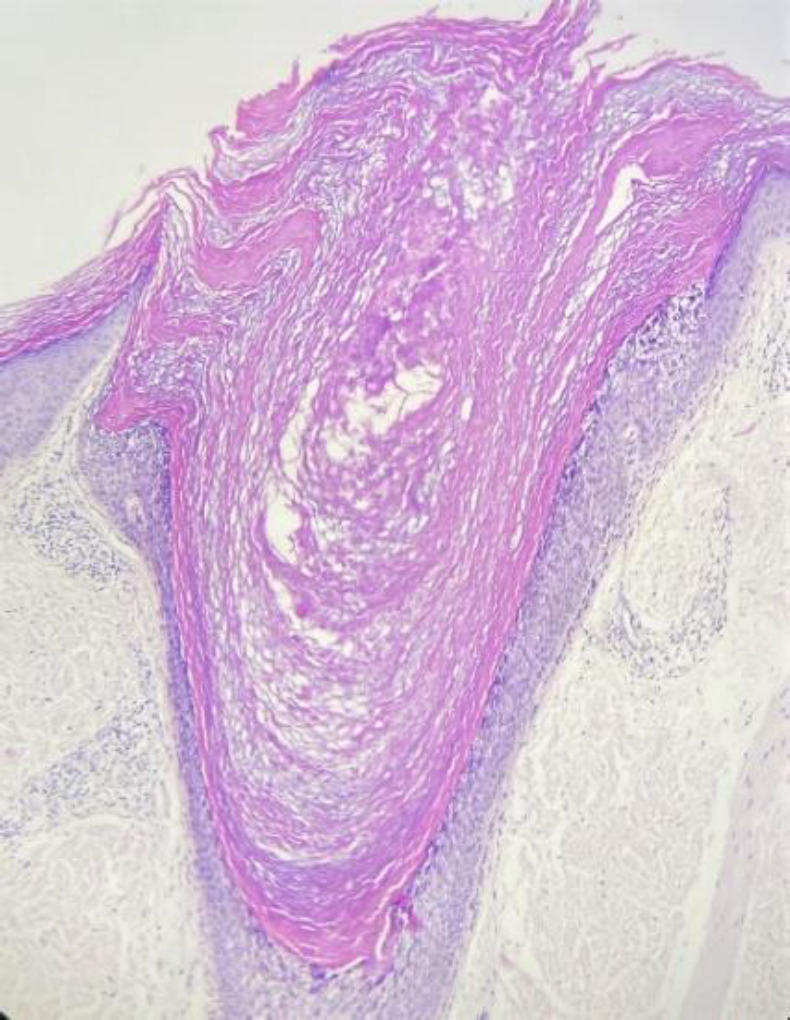
Comedo-like openings in follicular ostia

**Fig. 6:**
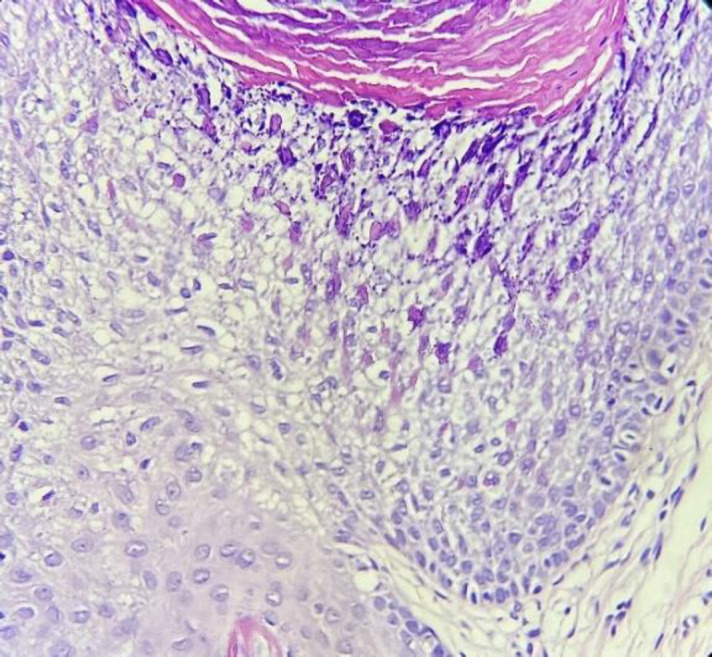
Epidermolytic hyperkeratosis in the follicular comedo-like wall

As a result, the histopathology of one of the papules of the thigh showed dilated follicular ostia (comedo-like openings) with orthokeratotic hyperkeratosis (Figures 3, 4, 5), columns of parakeratosis, cornoid flagellation, epidermolytic hyperkeratosis (Figure 6), and mild acanthosis on its wall. 

Based on the clinical presentation, which included linear unilateral persistent and asymptomatic lesions from 10 years ago, without any inflammation and scar-ring and only in the form of comedo-like brown-black follicular papules and histopathological findings, Nev-us comedonicus with follicular epidermolytic hyper-keratosis was diagnosed.

A topical keratolytic agent was prescribed. Alth-ough she was advised to return one month later to evaluate the response to treatment, the patient did not seek follow-up.

## Discussion

Kofmann, in 1895, was the first to define NC (1). It appears shortly after birth and mostly before the age of 10; however, late-onset cases have been reported (1). There is no gender or racial predilection (3). NC is one of the uncommon types of epidermal nevi, which can be a component of nevus comedonicus syndrome, a neurocutaneous disorder with skeletal, ocular, and central nervous system abnormalities (3).

NC can be distributed linearly, unilaterally, bilaterally, segmentally, or along blaschko lines (4). Clinical presentation is clusters of dilated follicular ostia with firm, black keratinous plugs, but the material cannot be brought out easily, unlike classical come-done (1, 2). 

Generally, it is located on the face with no symp-toms, followed by the trunk, neck, and upper extremity (1). It also affects palms, soles, and glans penis without hair follicles (1, 2).

In a pilosebaceous unit development, the intera-ction between fibroblast growth factor (FGF) and FGF receptor-2 (FGFR2) has the main role. One of the possible hypotheses in NC pathogenesis is oversti-mulation of FGFR2 signaling and increased expression of interleukin-1α (5); another candidate is ɣ-Secretase in the human hair follicle (6).

Histology reveals dilated follicular openings conta-ining keratinous debris devoid of hair shafts. Rarely epidermolytic hyperkeratosis (EHK) without any para- or dyskeratosis is present (7). EHK, an autosomal dominant disorder of cornification, mainly results from mutations in the genes encoding keratin 1 or 10 (8). EHK finding in histopathology has increased the thickness of the stratum corneum, hypergranulosis, intracellular vacuolization in stratum granulosum and stratum spinosum, and large, irregular keratohyalin granules in the granular cell layer (9).

Electron microscopy of NC lesions shows an increase in the number of Langerhans cells, tono-filaments in the upper layers of the stratum spinosum, as well as keratohyalin granules (10). The expression of cytokeratin in NC is similar to normal skin (11). Commonly filaggrin is often found in the granular layer of the epidermis. Still, in NC, it was found in all epidermal layers of close comedone, which suggests the role of filaggrin in the development of these lesions (11, 12).

EHK has been reported in several other diseases, including linear epidermal nevi**, **palmar keratoderma, seborrheic keratosis, acanthomas, and congenital ichth-yosiform erythroderma (13). EHK properties in NC are not a common finding and are rarely seen in association with each other (14). This accompaniment can lead to generalized EHK in children if a postzygotic mutation in germline cells occurs (5). We know, few cases of nevus comedonicus with epidermolytic hyperkeratosis (NCEHK) were reported (15).

Conservative treatments include emollients, topical corticosteroids, keratolytic agents like 12% ammonium lactate or salicylic acid, topical tazarotene with calcipotriene (1) topical benzoyl peroxide10% gel (15). There are also reports of improvement with surgical excision in localized NC (1). Lasers such as 2,940-nm erbium YAG, 1,450-nm diode, or 10,600-nm ultra pulsed CO_2_ are also used to treat NC (1). 

## Conclusion

Nevus comedonicus is a rare type of epidermal nevus. Histopathological findings of NC include dil-ated follicular openings containing keratinous debris devoid of hair shafts. EHK without any para- or dyskeratosis is rarely present.

An essential point about NC is considering possible correlations with other extracutaneous findings of the nevus comedonicus syndrome. If NC is not acco-mpanied by other extracutaneous manifestations in NC syndrome such as skeletal, ocular, and CNS disorders, it is a benign lesion in itself. It is important to report and follow up with patients because its treatment minimizes future disfiguring lesions. The study of the pathogenesis of NC enhances our understanding of the pathological mechanisms involved in the pilose-baceous unit.

Treatments available for NC include surgical removal, lasers, and topical treatments such as emol-lients, topical corticosteroids, keratolytic agent, and topical benzoyl peroxide10% gel.

## Conflict of Interest

The authors declared no conflict of interest.

## Funding

None.

## References

[1] Tchernev G, Ananiev J, Semkova K, Dourmishev LA, Schonlebe J, Wollina U (2013). Nevus comedonicus: an updated review. Dermatol Ther (Heidelb)..

[2] Nabai H, Mehregan AH (1973). Nevus comedonicus. A review of the literature and report of twelve cases. Acta Derm Venereol..

[3] Patrizi A, Neri I, Fiorentini C, Marzaduri S (1998). Nevus comedonicus syndrome: a new pediatric case. Pediatr Dermatol..

[4] Happle R (2010). The group of epidermal nevus syndromes Part I. Well defined phenotypes. J Am Acad Dermatol..

[5] Melnik B, Schmitz G (2008). FGFR2 signaling and the pathogenesis of acne. J Dtsch Dermatol Ges..

[6] Pan Y, Lin MH, Tian X, Cheng HT, Gridley T, Shen J (2004). gamma-secretase functions through Notch signaling to maintain skin appendages but is not required for their patterning or initial morphogenesis. Dev Cell..

[7] Schecter AK, Lester B, Pan TD, Robinson-Bostom L (2004). Linear nevus comedonicus with epidermolytic hyperkeratosis. J Cutan Pathol..

[8] DiGiovanna JJ, Bale SJ (1994). Epidermolytic hyperkeratosis: applied molecular genetics. J Invest Dermatol..

[9] Aloi FG, Molinero A (1987). Nevus comedonicus with epidermolytic hyperkeratosis. Dermatologica..

[10] Lee S, Nasemann T, Neufahrt A (1972). [Histogenesis of nevus comedonicus. Histochemical, light and electron microscopy studies]. Hautarzt..

[11] Kurokawa I, Nakai Y, Nishimura K, Hakamada A, Isoda K, Yamanaka K (2007). Cytokeratin and filaggrin expression in nevus comedonicus. J Cutan Pathol..

[12] Wollina U (1997). Histochemistry of the human hair follicle. Exs..

[13] Barsky S, Doyle JA, Winkelmann RK (1981). Nevus comedonicus with epidermolytic hyperkeratosis. A report of four cases. Arch Dermatol..

[14] Lookingbill DP, Ladda RL, Cohen C (1984). Generalized epidermolytic hyperkeratosis in the child of a parent with nevus comedonicus. Arch Dermatol..

[15] Zanniello R, Pilloni L, Conti B, Faa G, Rongioletti F (2019). Late-Onset Nevus Comedonicus With Follicular Epidermolytic Hyperkeratosis-Case Report and Review of the Literature. Am J Dermatopathol..

